# Spinal Cord Compression due to Pyothorax‐Associated Lymphoma: A Rare Presentation of EBV‐Positive Diffuse Large B‐Cell Lymphoma

**DOI:** 10.1155/cro/7073090

**Published:** 2026-06-10

**Authors:** Keita Yanagisawa, Junya Saito, Hiroshi Takahashi, Arata Nakajima, Koichi Nakagawa

**Affiliations:** ^1^ Department of Orthopaedic Surgery, Toho University Sakura Medical Center, Chiba, Japan, toho-u.ac.jp; ^2^ Department of Orthopaedic Surgery, Faculty of Medicine, University of Tsukuba, Ibaraki, Japan, tsukuba.ac.jp

## Abstract

**Background:**

Pyothorax‐associated lymphoma (PAL) is an Epstein–Barr virus (EBV)–positive diffuse large B‐cell lymphoma developing in the setting of long‐standing chronic pyothorax. Neurological involvement due to spinal invasion is extremely rare.

**Case Presentation:**

An 87‐year‐old woman with a remote history of pulmonary tuberculosis presented with rapidly progressive paraplegia. Imaging demonstrated chronic right pyothorax with a dorsal epidural mass compressing the spinal cord, continuous with a pleural lesion. A biopsy confirmed EBV‐positive diffuse large B‐cell lymphoma, consistent with PAL. Surgery was considered but declined by the patient and the family. Therefore, the patient was treated with corticosteroids followed by two cycles of R‐CHOP chemotherapy; however, no neurological improvement was observed.

**Conclusion:**

This case highlights a rare neurological manifestation of PAL. Clinicians should consider lymphoma infiltration in elderly patients with chronic pyothorax who develop new neurological deficits. Early MRI and biopsy are crucial for accurate diagnosis and management.

## 1. Introduction

Pyothorax‐associated lymphoma (PAL) is a rare Epstein–Barr virus (EBV)–positive diffuse large B‐cell lymphoma that arises in the setting of chronic pyothorax, often decades after artificial pneumothorax for tuberculosis [1, 2]. While PAL typically manifests as pleural masses, epidural invasion causing spinal cord compression is extremely uncommon.

## 2. Case Presentation

An 87‐year‐old woman with a remote history of pulmonary tuberculosis presented with progressive weakness in the lower limbs over 2 weeks. Neurological examination revealed paraplegia and sensory loss below the T8 level. No B symptoms, including fever, night sweats, or weight loss, were observed. Laboratory findings showed mild inflammatory changes without abnormal lymphocyte proliferation. The white blood cell count was mildly elevated (10,770/*μ*L), and the lactate dehydrogenase level was also slightly increased (382 U/L), whereas red blood cell and platelet counts were within normal limits.

Computed tomography (CT) demonstrated pleural calcification in the right hemithorax and a mass with peripheral calcification in the right thoracic cavity, suggestive of chronic pyothorax. A soft tissue lesion along the right posterior chest wall raised suspicion for a tumor arising in this setting (Figure 1).

**Figure 1:**
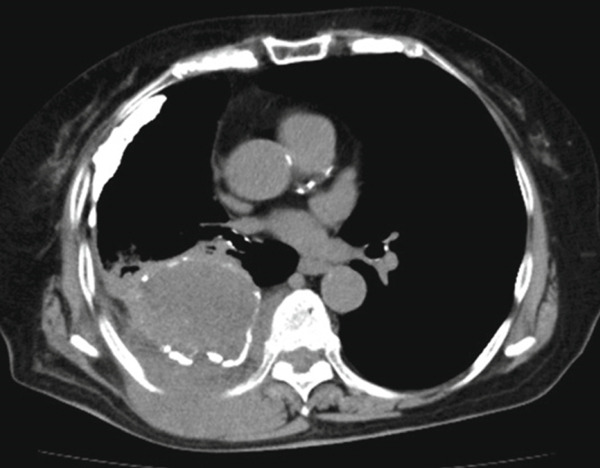
Noncontrast CT demonstrating pleural calcification in the right hemithorax, consistent with prior tuberculosis, and a mass with peripheral calcification in the right thoracic cavity, suggestive of chronic pyothorax. A soft tissue lesion is also noted along the right posterior chest wall, raising suspicion for a tumor associated with chronic pyothorax.

Contrast‐enhanced magnetic resonance imaging (MRI) revealed a right‐dominant dorsal epidural mass at the T6–T8 levels, extending from the posterior chest wall into the spinal canal through the intervertebral foramina and causing spinal cord compression. No signal changes suggestive of discitis, vertebral osteomyelitis, or vertebral body destruction were identified. These findings were highly suggestive of malignant lymphoma, particularly PAL (Figure 2).

**Figure 2:**
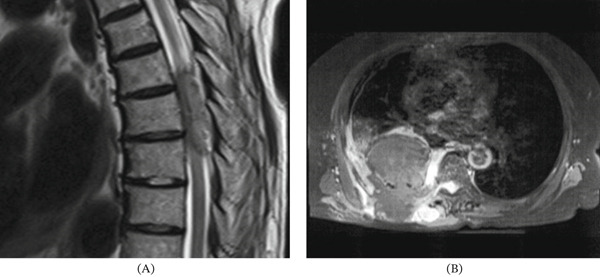
MRI demonstrating a right‐dominant epidural mass at the T6–T8 levels. (A) Sagittal T2‐weighted image showing the longitudinal extent of the lesion and spinal cord compression. (B) Axial gadolinium‐enhanced fat‐suppressed T1‐weighted image demonstrating a heterogeneously enhancing epidural mass extending into the spinal canal through the intervertebral foramina.

Differential diagnoses included metastatic spinal tumor, epidural abscess, and other malignant lymphomas. However, MRI demonstrated no vertebral body destruction or disc signal changes suggestive of infectious spondylitis. The epidural lesion showed heterogeneous contrast enhancement, favoring a neoplastic process rather than infection. Contrast‐enhanced CT from the cervical region to the pelvis revealed no apparent primary malignancy. In addition, calcified pleural thickening consistent with old tuberculosis and a calcified intrathoracic mass lesion suggestive of chronic pyothorax were identified in the right thoracic cavity. Surrounding chest wall masses were also observed. Based on these findings, PAL was strongly suspected. Soluble interleukin‐2 receptor was mildly elevated at 1140 U/mL (reference range: 145–519 U/mL).

CT‐guided biopsy of the pleural mass revealed diffuse proliferation of large atypical lymphoid cells. Immunohistochemical analysis was consistent with diffuse large B‐cell lymphoma. In situ hybridization for Epstein–Barr virus‐encoded RNA (EBER) was positive, supporting the diagnosis of PAL. Because the patient and family strongly preferred nonsurgical management from the time of admission, surgical decompression was not pursued. In addition, the lesion was considered potentially responsive to chemotherapy because malignant lymphoma was strongly suspected based on the imaging findings and clinical background. Therefore, systemic chemotherapy with R‐CHOP was selected after multidisciplinary discussion involving orthopedic surgeons, hematologists, radiologists, and pathologists. However, no neurological improvement was observed after two cycles of R‐CHOP chemotherapy. Follow‐up continued for approximately 2 months after treatment initiation, after which the patient discontinued outpatient follow‐up visits. The clinical course of the patient is summarized in Table 1.

**Table 1:** Timeline of the patient’s clinical course from symptom onset to follow‐up.

Time	Clinical course
3 months before admission	The patient developed back pain and discomfort in both lower extremities and received conservative treatment at a local orthopedic clinic.
Before admission	Progressive numbness and muscle weakness in both lower extremities resulted in gait disturbance.
Day 0	MRI demonstrated an epidural lesion causing spinal cord compression.
Hospital Day 3	CT‐guided biopsy was performed.
Hospital Day 12	Histopathological examination confirmed EBV‐positive diffuse large B‐cell lymphoma associated with PAL.
Hospital Day 20	R‐CHOP chemotherapy was initiated.
3 weeks later	A second cycle of R‐CHOP was administered.
2 months after treatment initiation	No neurological improvement was observed.
Thereafter	Follow‐up was discontinued.

## 3. Discussion

PAL is a rare subtype of diffuse large B‐cell lymphoma that develops in chronically inflamed pleural cavities, typically decades after tuberculous pyothorax or artificial pneumothorax therapy [1, 2]. Chronic inflammation and EBV infection are key pathogenic factors contributing to the malignant transformation of B cells [3, 4]. In the present case, EBER positivity confirmed EBV involvement, consistent with the typical features of PAL.

Although most PAL cases are localized to the pleura, extension into adjacent structures, including the spine, is exceedingly rare [5, 6]. In the nationwide review of 106 PAL cases by Nakatsuka et al. [1], only two patients presented with lower extremity paralysis due to spinal cord invasion . In addition, only a few Japanese case reports describing epidural extension of PAL have been published, whereas English‐language reports remain very limited. In our case, the tumor extended from the thoracic cavity into the epidural space through the intervertebral foramina, resulting in spinal cord compression.

Treatment strategies for PAL are not well‐established; however, chemotherapy, particularly R‐CHOP–based regimens, is commonly used as a standard approach [7, 8]. In cases presenting with spinal cord compression, additional treatment options such as surgical decompression, radiotherapy, and corticosteroid therapy may be considered depending on the patient′s general condition and extent of disease. In the present case, surgical intervention was considered; however, it was not performed due to the patient′s condition and family preference. The patient was managed with corticosteroids followed by R‐CHOP chemotherapy; however, no neurological improvement was observed. Comprehensive staging evaluations such as PET‐CT and bone marrow examination were not performed because treatment was initiated promptly due to progressive neurological deterioration. This clinical course highlights the difficulty in achieving neurological recovery once significant spinal cord compression has occurred. Furthermore, previous reports of PAL with spinal involvement remain limited, and the optimal management strategy has not been established. Accumulation of additional cases is needed to better understand the clinical course and to establish appropriate treatment strategies for this rare condition.

This case highlights a rare presentation of PAL with epidural extension causing spinal cord compression. Clinicians should be aware of this condition, particularly in patients with a history of chronic pyothorax, as early recognition may facilitate prompt diagnosis and appropriate management.

This case report was prepared in accordance with the CARE guidelines.

## 4. Conclusion

We presented a rare case of spinal cord compression caused by epidural extension of PAL. This case emphasizes the importance of considering lymphoma infiltration in elderly patients with chronic pyothorax who develop new neurological deficits.

## Funding

No funding was received for this manuscript.

## Disclosure

After using the AI tool, the authors reviewed and edited the content and take full responsibility for the final version.

## Ethics Statement

According to the institutional policy of Toho University, formal ethical approval is not required for single‐patient case reports. Verbal informed consent for publication was obtained from the patient′s family. Written informed consent could not subsequently be obtained because the family could no longer be contacted for documentation. All identifying information was anonymized to protect patient privacy. This study was conducted in accordance with institutional ethical standards and the Declaration of Helsinki.

## Conflicts of Interest

The authors declare no conflicts of interest.

## Supporting information


**Supporting Information** Additional supporting information can be found online in the Supporting Information section. CARE checklist completed according to the CARE reporting guideline for case reports.

## Data Availability

The data that support the findings of this study are available on request from the corresponding author. The data are not publicly available due to privacy or ethical restrictions.
