# Supplementary material for: Spinal Cord Compression due to Pyothorax‐Associated Lymphoma: A Rare Presentation of EBV‐Positive Diffuse Large B‐Cell Lymphoma
Source: Case Rep Orthop. 2026 Jun 10;2026:7073090. doi: 10.1155/cro/7073090 (PMC13254235; doi:10.1155/cro/7073090)
Supplement: Supplementary file 1 — Supporting Information Additional supporting information can be found online in the Supporting Information section. CARE checklist completed according to the CARE reporting guideline for case reports. [file CRO-2026-7073090-s001.docx]

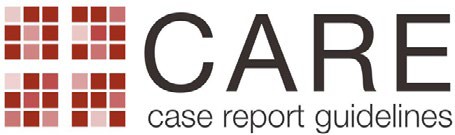

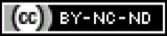

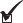
**CARE Checklist of information to include when writing a case report**

| **Topic** | **Item No** | **Checklist item description** | **Reported on Page Number/Line Number** | **Reported on Section/Paragraph** |
| --- | --- | --- | --- | --- |
| Title | 1 | The diagnosis or intervention of primary focus followed by the words “case report” | Page 1 | Title |
| Key Words | 2 | 2 to 5 key words that identify diagnoses or interventions in this case report, including "case report" | Page 1 | Key words |
| Abstract  (Structured summary) | 3a | Background: state what is known and unknown; why the case report is unique and what it adds to existing literature. | Page 3 | Abstract |
|  | 3b | Case Description: describe the patient’s demographic details, main symptoms, history, important clinical findings, the main diagnosis, interventions, outcomes and follow-ups. | Page 3 | Abstract |
|  | 3c | Conclusions: summarize the main take-away lesson, clinical impact and potential implications. | Page 3 | Abstract |
| Introduction | 4 | One or two paragraphs summarizing why this case is unique **(may include references)** | Page 3 | Introduction |
| Patient Information | 5a | De-identified patient specific information | Page 3 | Case Presentation |
|  | 5b | Primary concerns and symptoms of the patient | Page 3 | Case Presentation |
|  | 5c | Medical, family, and psycho-social history including relevant genetic information | Page 3 | Case Presentation |
|  | 5d | Relevant past interventions with outcomes | Page 3 | Case Presentation |
| Clinical Findings | 6 | Describe significant physical examination (PE) and important clinical findings | Page 3 | Case Presentation |
| Timeline | 7 | Historical and current information from this episode of care organized as a timeline | Table 1 | Table 1 |
| Diagnostic Assessment | 8a | Diagnostic testing (such as PE, laboratory testing, imaging, surveys). | Page 3-4 | Case Presentation |
|  | 8b | Diagnostic challenges (such as access to testing, financial, or cultural) | N/A | N/A |
|  | 8c | Diagnosis (including other diagnoses considered) | Page 4 | Case Presentation |
|  | 8d | Prognosis (such as staging in oncology) where applicable | Page 5 | Discussion |
| Therapeutic Intervention | 9a | Types of therapeutic intervention (such as pharmacologic, surgical, preventive, self-care) | Page 4 | Case Presentation |
|  | 9b | Administration of therapeutic intervention (such as dosage, strength, duration) | Page 4 | Case Presentation |
|  | 9c | Changes in therapeutic intervention (with rationale) | Page 4 | Case Presentation |

| Follow-up and Outcomes | 10a | Clinician and patient-assessed outcomes (if available) | Page 4-5 | Case Presentation |
| --- | --- | --- | --- | --- |
|  | 10b | Important follow-up diagnostic and other test results | Page 4 | Case Presentation |
|  | 10c | Intervention adherence and tolerability (How was this assessed?) | N/A | N/A |
|  | 10d | Adverse and unanticipated events | N/A | N/A |
| Discussion | 11a | A scientific discussion of the strengths AND limitations associated with this case report | Page 5 | Discussion |
|  | 11b | Discussion of the relevant medical literature **with references** | Page 4-5 | Discussion |
|  | 11c | The scientific rationale for any conclusions (including assessment of possible causes) | Page 4-5 | Discussion |
|  | 11d | The primary “take-away” lessons of this case report (without references) in a one paragraph conclusion | Page 5 | Conclusion |
| Patient Perspective | 12 | The patient should share their perspective in one to two paragraphs on the treatment(s) they received | N/A | N/A |
| Informed Consent | 13 | Did the patient give informed consent? Please provide if requested | Yes ■ | No |
